# Encouraging Adults at Risk for Type 2 Diabetes to Enroll in Diabetes Prevention Programs Through a Media Campaign in Hawai’i: Cross-Sectional Study

**DOI:** 10.2196/90880

**Published:** 2026-06-18

**Authors:** Kara Saiki, Eunjung Lim, Blythe Nett, Gail Ogawa, Lance K Ching, Riana M Kawasaki, Nianest Alers Barreto, Katherine Inoue, Lola H Irvin, Kiana R Kaupiko, L Brooke Keliikoa, Meghan D McGurk

**Affiliations:** 1 Healthy Hawai'i Evaluation Team, Department of Public Health Sciences, University of Hawai'i at Mānoa, 1960 East-West Rd, Biomed D-210, Honolulu, HI, 96822, United States, 1 808-956-5505; 2 Department of Quantitative Health Sciences, John A Burns School of Medicine, University of Hawai'i at Mānoa, Honolulu, HI, United States; 3 Chronic Disease Prevention and Health Promotion Division, Hawai'i State Department of Health, Honolulu, HI, United States

**Keywords:** prediabetes, diabetes prevention program, public health, intention, motivation, media campaign

## Abstract

**Background:**

The National Diabetes Prevention Program (DPP) is an evidence-based intervention proven to delay or prevent progression to type 2 diabetes, yet most at-risk people do not enroll. In Hawai’i, Native Hawaiian and Other Pacific Islander (NHOPI) and Filipino adults experience disproportionately high rates of prediabetes and diabetes but have low DPP enrollment. From July to October 2024, the Hawai’i State Department of Health launched Beat Diabetes, a statewide media campaign encouraging DPP enrollment among at-risk adults, with a focus on NHOPI and Filipino communities.

**Objective:**

This evaluation assessed whether campaign exposure was associated with self-reported likelihood of joining a DPP among Hawai’i adults at risk for diabetes, particularly NHOPI or Filipino adults.

**Methods:**

A postcampaign cross-sectional online survey was conducted from October to December 2024, with Hawai’i residents aged 35-64 years who reported at least 1 diabetes risk factor. NHOPI or Filipino adults were oversampled to determine campaign effectiveness among the target audience. The survey measured self-reported likelihood of joining a lifestyle change program (main outcome), campaign recall (main exposure), demographic characteristics, diabetes risk factors, and beliefs that could affect DPP enrollment likelihood, including intrinsic motivation, perceived inevitability of developing diabetes, and perceived health benefits of DPP participation. Three general linear regression models examined the association between campaign exposure and DPP enrollment likelihood ratings, adjusted for demographic characteristics, diabetes risk factors, and belief variables. A sensitivity analysis among just those diagnosed with prediabetes was conducted.

**Results:**

A total of 860 adults completed the survey, with 34.7% (298/860) and 12.2% (105/860) self-identifying as NHOPI and Filipino, respectively. In total, 40% (346/860) reported campaign exposure. Exposed individuals had higher mean DPP enrollment likelihood ratings and higher inevitability belief scores than those not exposed. A large proportion of exposed respondents reported that enrolling in a DPP would “improve their health a lot.” No significant differences in campaign exposure were observed across ethnicities. All 3 regression models showed a significant positive association between campaign exposure and DPP enrollment likelihood ratings. In the final adjusted model controlling for all covariates, significant predictors included campaign exposure (*β*=.52, *P*<.001), male gender (*β*=.34, *P*=.01), residence outside Honolulu County (*β*=.31, *P*=.02), motivation index scores (*β*=.38, *P*<.001), inevitability belief (*β*=.20*, P*<.001), and the belief that DPP improves health “a little” (*β*=.76, *P*<.001) or “a lot” (*β*=1.63, *P*<.001). The sensitivity analysis among those diagnosed showed exposure was not associated with likelihood ratings (*β*=.30, *P*=.27).

**Conclusions:**

Campaign exposure was associated with higher ratings of likelihood to join a DPP among at-risk adults with no prediabetes diagnosis. Perceived positive health impact of DPP participation was the strongest contributor to likelihood ratings. Campaigns aiming to increase awareness of DPP and intentions to join should promote DPP effectiveness and the urgency of preventative actions.

## Introduction

### Background

In the United States, approximately 97.6 million adults have prediabetes [1], placing more than one-third of the adult population at risk for developing type 2 diabetes. Although the statewide prevalence of diagnosed prediabetes in Hawai’i is lower than the national estimate (14.9% vs 38%) [1,2], disaggregated data reveal notable differences among racial and ethnic groups. Native Hawaiian (17.2%), Other Pacific Islander (16.9%), and Filipino (17.3%) adults have higher prevalence of diagnosed prediabetes than non-Hispanic White (9.0%) adults [2]. These groups, along with non–Hispanic Black and Hispanic adults, are recognized nationally as having elevated risk for type 2 diabetes [3].

Established risk factors for prediabetes and diabetes include being older than 40 years, having a family history of diabetes, having high blood pressure, physical inactivity, a history of gestational diabetes, and being overweight [4,5]. In 2021, the US Preventive Services Task Force lowered the recommended prediabetic screening age for adults who are overweight or obese from 40 to 35 years, citing these risk factors as the strongest predictors for progressing to diabetes [6]. Identifying individuals at high risk for prediabetes and intervening early is critical to preventing progression to type 2 diabetes.

The National Diabetes Prevention Program (DPP) is an evidence-based lifestyle intervention that can reduce the risk of progressing from prediabetes to type 2 diabetes by up to 58% [7]. Despite the success of the program, less than 1% of US adults with prediabetes enroll in the program [8]. Enrollment data show that certain groups such as Native Hawaiian and Other Pacific Islander (NHOPI) and Filipino adults are disproportionately affected by prediabetes [9] and yet enroll in DPPs at lower rates than Hispanic, non–Hispanic Black, and non–Hispanic White adults [8].

Multiple factors contribute to low enrollment. Extrinsic barriers include program cost, limited time to participate, long wait periods before enrollment, lack of online options, and competing health conditions [10-17]. Intrinsic barriers also play a role. Low autonomous motivation, defined as low internal drive to adopt healthier behaviors [18], and belief that diabetes is inevitable due to family history may reduce individuals’ willingness to engage in prevention efforts [19,20]. Together, these barriers can hinder adults with prediabetes from taking steps to prevent disease progression.

### Campaign Development

In 2024, the Hawai’i State Department of Health (HDOH) conducted focus groups to better understand why adults diagnosed with prediabetes or at high risk for prediabetes do not engage in lifestyle changes and what messages might motivate enrollment in a DPP. Focus groups were segmented by the largest racial and ethnic groups in Hawai’i: Native Hawaiian, Other Pacific Islander, Filipino, and Other (White and Japanese) adults with focused recruitment of NHOPI or Filipino adults due to their high rates of prediabetes. Across groups, most participants reported that they did not have time to prioritize their health. Some participants felt little urgency to join a lifestyle change program, stating that they would be more likely to seek help only after receiving a diabetes diagnosis. Others were uncertain about how to start making a change but responded positively to messages that were direct and encouraging. Participants preferred campaign materials featuring people from diverse backgrounds, including NHOPI or Filipino adults.

Guided by these findings, the HDOH developed the Beat Diabetes media campaign to increase urgency around diabetes prevention and provide a clear call to action by joining DPP, a lifestyle change program. The campaign aimed to increase DPP enrollment overall, particularly for NHOPI or Filipino adults at risk for prediabetes and diabetes, by addressing intrinsic barriers such as low motivation and perceived inevitability of diabetes. The campaign consisted of 6 still advertisements and 1 video advertisement featuring NHOPI or Filipino adults delivering messages such as: “I’m not waiting. I’m preventing diabetes. Join the program.” and “Tomorrow’s a hard nope. I’m preventing diabetes today. Join the program.” (Multimedia Appendix 1). Each advertisement contained a link to the landing page for the Beat Diabetes website, which provided more information about prediabetes, diabetes risk test, frequently asked questions, and information on how to enroll in a DPP [21]. The campaign ran from July to October 2024 across statewide television, radio, digital platforms, print outlets, and mall posters.

To evaluate the effect of the campaign on self-reported likelihood of joining a lifestyle change program, particularly among the campaign’s target audience of NHOPI or Filipino adults at risk for diabetes, the HDOH collaborated with the Healthy Hawai’i Evaluation Team (HHET) to develop a postcampaign cross-sectional survey. A market research firm was contracted to administer the survey.

## Methods

### Study Design

This cross-sectional study followed the STROBE (Strengthening the Reporting of Observational Studies in Epidemiology) reporting guideline and checklist to draft this manuscript (Checklist 1) [22] and the CHERRIES (Checklist for Reporting Results of Internet E-Surveys) statement to report on web-based surveys (Checklist 2) [23].

### Survey Development

The survey was developed by the HDOH and HHET teams, and programming of the screening and core survey questions was checked and revised by the market research firm. The online survey was pretested with 25 eligible respondents before fielding. It took approximately 10‐12 minutes to complete and was composed of 7 screening questions and approximately 50 core questions in English. The survey could be viewed multiple ways: on a mobile phone showing 1 question per screen or on a desktop computer displaying multiple questions at once for a total of 24 screens.

### Participant Recruitment

Data collection occurred between October and December 2024. A convenience sample of adults aged 35‐64 years was recruited from 3 proprietary statewide market research consumer panels. The market research firm emailed eligible panelists an invitation to complete a closed online survey via a secured survey link, following the conclusion of the Beat Diabetes campaign. Consumer panels were selected over other sampling methods as they are a more cost-effective method to evaluate health department media campaigns to provide timely feedback for campaign improvement [24]. Inclusion criteria included residency in Hawai’i for at least 6 months and a diagnosis of either prediabetes or borderline diabetes, or at least 1 recognized prediabetes risk factor as determined by a diabetes risk test. Individuals with a prior diabetes diagnosis were excluded, as they are ineligible for DPP enrollment and may instead join a diabetes self-management education and support program. A minimum of 700 responses were required, with a goal of 900 responses determined by the budget set aside for recruitment. NHOPI and Filipino adults were oversampled to ensure that they made up 50% of the end sample, a large enough sample size to examine campaign recall and effectiveness within the campaign’s target audience.

### Ethical Considerations

This evaluation was reviewed by the HDOH Institutional Review Board, who confirmed it qualified as nonhuman subjects research under the revised Common Rule of 2018. Informed consent identified the purpose of the survey and who was conducting the investigation, identified the market research firm assisting with data collection, and gave details on the survey length and time, confidentiality and deidentification of data, and how participation was voluntary before eligible participants accessed the online survey. All survey responses were kept confidential on password-protected computers and deidentified prior to analysis. Incentives were provided by the marketing firm via each respondent’s email address and ranged from points to gift cards valued at US $10, depending on the panel. The marketing firm retained email addresses in a separate working file used solely for incentive distribution, with no link to survey responses so that no personal identifier could be tracked.

### Survey Measures

The full postsurvey instrument is provided in Multimedia Appendix 2. Survey measures included the following:

1. DPP enrollment likelihood rating: The main evaluation outcome was participants’ self-reported likelihood of joining a lifestyle change program. Participants were given a short description of such a program and asked, “How likely are you to consider joining a lifestyle change program such as *Beat Diabetes*?” Responses were recorded on a 7-point Likert scale ranging from 1 (*not at all likely*) to 7 (*very likely*). DPP enrollment likelihood ratings were treated as continuous in all analyses.
2. Campaign exposure: Campaign exposure was defined by self-reported recall of any of the campaign advertisements. Participants were shown 4 different still advertisements and 1 video advertisement and asked whether they had seen or heard each. Advertisements were rotated to reduce response bias. If participants had not seen or heard any of these advertisements, they were shown 2 additional still advertisements in a single question. Recall of any of the 7 advertisements was coded as exposure to the campaign.
3. Demographic characteristics: Participants reported their age, gender (woman and man), education, household income, and county of residence (Honolulu County and other counties). Health insurance status and type (private, Medicare, Medicaid, and TRICARE/other) were also collected. Participants reported the group that best represented their race or ethnicity. Race or ethnicity was coded into 3 categories: Filipino, NHOPI, and Other. Participants indicating Filipino alone or along with another race or ethnicity were categorized as Filipino. Participants reporting at least one of the following ethnicities alone or with any other race or ethnicity (ie, multiethnic participants)—Native Hawaiian or Part Hawaiian and Other Pacific Islander (including Samoan, Tongan, Guamanian or Chamorro, Fijian, and peoples of Micronesia and Melanesia)—were categorized as NHOPI. All other racial and ethnic groups were categorized as “Other.”
4. Diabetes risk factors: Screening questions were used to identify diabetes risk factors, including self-perceived overweight status, physical inactivity (less than 3 times per week), hypertension diagnosis, and prediabetes diagnosis. Family history risk was determined by responses to “My family members or relatives have diabetes” in the screening question and “Does your family have a history of diabetes?” in the main survey. Gestational diabetes risk combined 2 items: “I have given birth to a baby that weighed more than 9 pounds” and “I had gestational diabetes in a past pregnancy.” Only those indicating woman as gender were asked about gestational diabetes risk.
5. Motivation index score: Four items adapted from the Treatment Self-Regulation Questionnaire [18] assessed participants’ autonomous motivation to prevent diabetes. Participants rated each statement on a 7-point scale from 1 (*not at all true*) to 7 (*very true*). Each statement started with, “The reason I would take steps to help prevent diabetes is that...” (1) “It is very important for being as healthy as possible,” (2) “I personally believe it is the best thing for my health,” (3) “I feel that I want to take responsibility for my own health,” and (4) “It is consistent with my life goals.” All items were treated as continuous, with interitem correlations ranging from 0.54 to 0.72. The 4 items were averaged to create a motivation index score for each participant. The scale demonstrated good reliability with a Cronbach α of 0.86 (95% CI 0.8‐0.9).
6. Inevitability belief: Participants’ perceptions of the inevitability of developing diabetes were measured using the statement, “It is only a matter of time before I get diabetes,” rated on the same 7-point scale for the motivation items. Scores were treated as continuous.
7. Perceived DPP effectiveness: Participants’ beliefs about the effectiveness of DPP were measured with the question, “If you joined a lifestyle program such as Beat Diabetes, do you think your health would*...decline, stay the same, improve a little,* or *improve a lot*.” For analyses, *decline* and *stay the same* responses were combined to indicate negative beliefs about the effectiveness of DPP.

### Data Integrity

Data quality was continuously monitored throughout collection. Through the market research firm’s data-cleaning protocols, surveys that took less than 5 minutes to complete were excluded from the completed surveys, as were those with duplicate IP addresses. Completeness of each survey was monitored by the market research firm and the number of completed surveys was reported in weekly field status updates alongside numbers of emailed contacts, those ineligible after screening, and surveys abandoned postscreening questions. Respondents were not able to review or change their answers once they submitted the survey. A final data cleaning was done to remove responses suspected to be bots due to open-ended responses that were nonsensical or inconsistent in their responses to multiple questions. The remaining responses were included in analysis.

### Data Analysis

Descriptive statistics were reported as frequencies and percentages for categorical variables and as means and standard deviations for continuous variables. Bivariate analyses were conducted to examine potential associations between campaign exposure, DPP enrollment likelihood ratings, and covariates using 2-sample *t* tests or chi-square tests, as appropriate. Missing data for gender and education were excluded from analysis.

Three general linear regression models were used to assess the association between campaign exposure and DPP enrollment likelihood ratings, controlling for covariates. Model 1 included demographic variables only, model 2 added diabetes risk factors to model 1, and model 3 further adjusted for motivation index score, inevitability belief, and perceived DPP effectiveness. Graphical tests were conducted to assess model assumptions. Because slight deviations from normality were observed, sensitivity analyses using ordered logistic regression (analyses not shown) were conducted to identify potential deviations from the general linear regression results. No significant deviations were detected and thus general linear regression models are presented. Multicollinearity among independent variables was evaluated using the variance inflation factor. Subgroup analyses were conducted to examine race in a disaggregated manner using model 3 stratified by race or ethnicity. A sensitivity analysis was also conducted using model 3 among participants diagnosed with prediabetes to evaluate consistency with the overall findings. All analyses were conducted using R (version 4.5.1; R Core Team). A *P* value less than .05 was considered statistically significant.

## Results

### Final Sample

**Figure 1.**
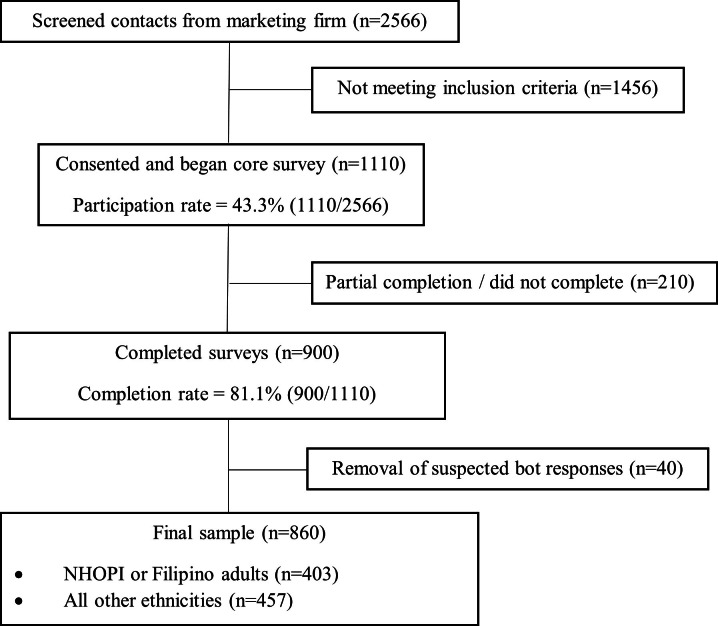
Flow diagram of sample development for a cross-sectional study of associations between campaign exposure and ratings of likelihood to join a National Diabetes Prevention Program among adults at risk for diabetes. NHOPI: Native Hawaiian and Other Pacific Islander.

A total of 2566 panel participants responded to the email invitation and were screened. Of these, 1456 were excluded for not meeting inclusion criteria and 210 were excluded for incomplete surveys, resulting in 900 completed surveys. An additional 40 responses were removed due to suspected bot activity, yielding a final analytic sample of 860 respondents (Figure 1).

### Sample Characteristics

The mean age was 49.2 (SD 8.5 years), with 64.7% (556/860) identifying as women, reflecting an overrepresentation of women compared with the state’s demographic composition [25]. Approximately half the sample, or 46.9% (403/860), self-identified as NHOPI or Filipino adults. Most participants reported a family history of diabetes (570/860, 66.3%) or perceived themselves as overweight (455/860, 52.9%). Despite all participants being classified as at risk based on screening criteria, only 22.7% (195/860) reported a prior diagnosis of prediabetes (Table 1).

**Table 1.** Participant characteristics by overall sample and those exposed and not exposed to the media campaign^a^.

Variable	Overall (N=860)	Exposed (n=346)	Not exposed (n=514)	*P* value^b^
Age (years), mean (SD)	49.2 (8.5)	49.4 (8.6)	49.0 (8.5)	.48
Gender, n (%)				.40
Woman	556 (64.7)	217 (39.0)	339 (61.0)	
Man	293 (34.1)	123 (42.0)	170 (58.0)	
Missing^c^	11 (1.3)	6	5	
Race or ethnicity, n (%)				.22
Filipino	105 (12.2)	34 (32.4)	71 (67.6)	
NHOPI^d^	298 (34.7)	123 (41.3)	175 (58.7)	
Other	457 (53.1)	189 (41.4)	268 (58.6)	
Education, n (%)				.16
HS^e^ or lower	151 (17.7)	50 (33.1)	101 (66.9)	
Some college	279 (32.6)	113 (40.5)	166 (59.5)	
College graduate	263 (30.8)	117 (44.5)	146 (55.5)	
Post college graduate	162 (18.9)	65 (40.1)	97 (59.9)	
Missing^c^	5 (0.6)	1	4	
Household income (US dollars), n (%)				.052
<$25,000	105 (12.2)	40 (38.1)	65 (61.9)	
$25,000-$50,000	148 (17.2)	44 (29.7)	104 (70.3)	
$50,001-$100,000	260 (30.2)	118 (45.4)	142 (54.6)	
$100,001-$150,000	173 (20.1)	75 (43.4)	98 (56.6)	
>$150,000	133 (15.5)	51 (38.3)	82 (61.7)	
Don’t know/missing	41 (4.8)	18 (43.9)	23 (56.1)	
Insurance type, n (%)				.13
Private	514 (59.8)	222 (43.2)	292 (56.8)	
Medicare	62 (7.2)	23 (37.1)	39 (62.9)	
Medicaid	151 (17.6)	57 (37.7)	94 (62.3)	
TRICARE/Other	89 (10.3)	33 (37.1)	56 (62.9)	
None/Missing	44 (5.1)	11 (25.0)	33 (75.0)	
County, n (%)				.02
Honolulu County	631 (73.4)	239 (37.9)	392 (62.1)	
All other counties	229 (26.6)	107 (46.7)	122 (53.3)	
Diabetes risk factors, n (%)				
Family history				<.001
Yes	570 (66.3)	257 (45.1)	313 (54.9)	
No	290 (33.7)	89 (30.7)	201 (69.3)	
Overweight perception				.03
Yes	455 (52.9)	199 (43.7)	256 (56.3)	
No	405 (47.1)	147 (36.3)	258 (63.7)	
Not physically active (<3 times per week)				.27
Yes	319 (37.1)	136 (42.6)	183 (57.4)	
No	541 (62.9)	210 (38.8)	331 (61.2)	
Gestational diabetes				.56
Yes	78 (9.1)	29 (37.2)	49 (62.8)	
No	782 (90.9)	317 (40.5)	465 (59.5)	
Diagnosed with high blood pressure				.83
Yes	237 (27.6)	94 (39.7)	143 (60.3)	
No	623 (72.4)	252 (40.4)	371 (59.6)	
Diagnosed with prediabetes				.04
Yes	195 (22.7)	91 (46.7)	104 (53.3)	
No	665 (77.3)	255 (38.3)	410 (61.7)	
Motivation index, mean (SD)	6.3 (0.9)	6.4 (0.8)	6.3 (1.0)	.26
Inevitability belief, mean (SD)	3.4 (2.1)	3.7 (2.2)	3.2 (2.0)	<.001
Perceived DPP^f^ effectiveness, n (%)				.04
Decline or stay the same	99 (11.5)	35 (35.4)	64 (64.6)	
Improve a little	302 (35.1)	110 (35.8)	192 (64.2)	
Improve a lot	364 (42.3)	167 (36.4)	197 (63.6)	
Don’t know	95 (11.0)	34 (45.9)	61 (54.1)	
DPP enrollment likelihood ratings, mean (SD)	3.6 (1.9)	4.1 (2.0)	3.2 (1.8)	<.001

a Row percentages are used to compare Exposed and Not exposed.

b Two-sample *t* test or chi-square test was used.

c Missing on gender and education were excluded from analyses.

d NHOPI: Native Hawaiian and Other Pacific Islander.

e HS: High school.

f DPP: National Diabetes Prevention Program.

### Sample Characteristics by Campaign Exposure

Table 1 also presents participant characteristics stratified by campaign exposure. Overall, 40.2% (346/860) of the sample reported exposure to the campaign. Among the 298 NHOPI or 105 Filipino participants, 123 (41.3%) and 34 (32.4%) reported being exposed to the campaign, respectively. Exposure to the campaign did not differ significantly across NHOPI, Filipino, and other racial or ethnic participant groups.

Significant differences between those exposed and not exposed to the campaign were observed by county of residence, specific diabetes risk factors, inevitability belief, and perceived DPP effectiveness. A smaller proportion of exposed participants resided in Honolulu County (239/631, 37.1%) compared with those not exposed (392/631, 62.1%; *P*=.02). Additionally, exposed participants were less likely to report a family history of diabetes (257/570, 45.1% vs 313/570, 54.9%; *P<*.001), perceive themselves as overweight (199/455, 45.1% vs 256/455, 56.3%; *P*=.03), or have been previously diagnosed with prediabetes (91/195, 46.7% vs 104/195, 53.3%; *P*=.04). Participants exposed to the campaign reported higher mean likelihood ratings for joining a DPP than those not exposed (mean 4.1, SD 2.0 vs mean 3.2, SD 1.8; *P*<.001) and had significantly higher mean scores for inevitability belief (mean 3.7, SD 2.2 vs mean 3.2, SD 2.0; *P*<.001). Fewer exposed participants believed that participation in a program such as Beat Diabetes would *improve* their health *a lot* (167/364, 42.3% vs 197/364, 63.6%; *P=*.04).

### Linear Regression Models on DPP Enrollment Likelihood Rating

Table 2 presents results from the 3 linear regression models. All models showed a significant positive effect of campaign exposure. In model 1, controlling for demographic characteristics, campaign exposure (β=.76, *P*<.001), age (β=−.02, *P*=.01), and insurance type (*P*=.02) were significantly associated with likelihood of joining a DPP.

**Table 2.** Linear regression models predicting National Diabetes Prevention Program enrollment likelihood ratings by campaign exposure, controlling for demographic characteristics, diabetes risk factors, and intrinsic factors.

	Model 1: demographic characteristics				Model 2: diabetes risk factors				Model 3: intrinsic factors			
Variable	β	SE	95% CI	*P* value	β	SE	95% CI	*P* value	β	SE	95% CI	*P* value
Intercept	4.50	0.54	3.43 to 5.57	<.001	3.20	0.58	2.05 to 4.34	<.001	-.55	0.68	-1.88 to 0.78	.42
Campaign exposure (ref^a^: no)				<.001				<.001				<.001
Yes	.76	0.13	0.50 to 1.03		.67	0.13	0.41 to 0.93		.52	0.12	0.28 to 0.75	
Age (years)	-.02	0.01	-0.04 to 0.00	.01	-.01	0.01	-0.03 to 0.01	.19	-.01	0.01	-0.03 to 0.00	.15
Gender (ref: women)				.16				.049				.01
Man	.20	0.14	-0.08 to 0.47		.28	0.14	0.00 to 0.56		.34	0.13	0.09 to 0.59	
Race/ethnicity (ref: Filipino)				.15				0.43				.19
NHOPI^b^	-.04	0.22	-0.47 to 0.40		.02	0.22	-0.41 to 0.44		.15	0.2	-0.24 to 0.54	
Other	-.29	0.21	-0.70 to 0.12		-.17	0.21	-0.57 to 0.24		-.10	0.19	-0.47 to 0.27	
Education (ref: HS^c^ or lower)				.27				0.29				.13
Some college	.25	0.2	-0.15 to 0.64		.14	0.2	-0.25 to 0.52		.14	0.18	-0.21 to 0.49	
College graduate	.23	0.22	-0.20 to 0.65		.17	0.21	-0.25 to 0.59		.15	0.19	-0.23 to 0.53	
Post college graduate	.47	0.24	0.00 to 0.95		.44	0.24	-0.03 to 0.91		.47	0.22	0.05 to 0.90	
Household income, US dollars (ref: <$25,000)				.53				.75				.85
$25,000-$50,000	-.24	0.26	-0.74 to 0.26		-.10	0.25	-0.59 to 0.39		-.11	0.23	-0.55 to 0.34	
$50,001-$100,000	-.37	0.25	-0.86 to 0.13		-.24	0.25	-0.72 to 0.25		-.27	0.22	-0.71 to 0.17	
$100,001-$150,000	-.24	0.28	-0.79 to 0.31		-.13	0.28	-0.67 to 0.41		-.27	0.25	-0.76 to 0.22	
>$150,001	-.37	0.3	-0.95 to 0.21		-.26	0.29	-0.83 to 0.32		-.29	0.27	-0.81 to 0.23	
Don’t know/missing	-.68	0.37	-1.41 to 0.06		-.52	0.37	-1.24 to 0.21		-.29	0.33	-0.95 to 0.36	
Insurance type (ref: private)				.02				.22				.27
Medicare	.00	0.27	-0.54 to 0.54		-.01	0.27	-0.54 to 0.52		.17	0.25	-0.31 to 0.65	
Medicaid	-.50	0.22	-0.93 to -0.08		-.32	0.21	-0.74 to 0.10		-.34	0.2	-0.72 to 0.05	
TRICARE/Other	-.18	0.22	-0.62 to 0.25		-.03	0.22	-0.46 to 0.40		.05	0.2	-0.35 to 0.44	
None/missing	-.90	0.32	-1.54 to -0.27		-.66	0.32	-1.29 to -0.03		-.24	0.29	-0.81 to 0.33	
County (ref: Honolulu County)				.09				.17				.02
All other counties	.26	0.15	-0.04 to 0.56		.21	0.15	-0.09 to 0.50		.31	0.14	0.05 to 0.58	
Diabetes risk factors												
Family history	N/A^d^	N/A	N/A	N/A	.33	0.14	0.05 to 0.61	.02	.11	0.13	-0.15 to 0.37	.41
Overweight perception	N/A	N/A	N/A	N/A	.47	0.14	0.19 to 0.75	.001	.19	0.13	-0.07 to 0.45	.15
Not physically active (<3 x per week)	N/A	N/A	N/A	N/A	.28	0.14	0.00 to 0.55	.047	.19	0.13	-0.06 to 0.44	.14
Gestational diabetes	N/A	N/A	N/A	N/A	.15	0.23	-0.31 to 0.60	.53	.03	0.21	-0.39 to 0.44	.90
Diagnosed with high blood pressure	N/A	N/A	N/A	N/A	.01	0.15	-0.28 to 0.31	.94	-.11	0.14	-0.37 to 0.16	.43
Diagnosed with prediabetes	N/A	N/A	N/A	N/A	.37	0.16	0.05 to 0.69	.02	.16	0.15	-0.14 to 0.45	.30
Motivation index	N/A	N/A	N/A	N/A	N/A	N/A	N/A	N/A	.38	0.07	0.25 to 0.51	<.001
Inevitability belief	N/A	N/A	N/A	N/A	N/A	N/A	N/A	N/A	.20	0.03	0.14 to 0.26	<.001
Perceived DPP^e^ effectiveness (ref: Decline/stay the same)				N/A				N/A				<.001
Don’t know	N/A	N/A	N/A		N/A	N/A	N/A		.38	0.25	-0.11 to 0.87	
Improve a little	N/A	N/A	N/A		N/A	N/A	N/A		.76	0.2	0.36 to 1.16	
Improve a lot	N/A	N/A	N/A		N/A	N/A	N/A		1.63	0.2	1.23 to 2.03	

a Ref: reference category.

b NHOPI: Native Hawaiian and Other Pacific Islander.

c HS: high school.

d N/A: not applicable.

e DPP: National Diabetes Prevention Program.

Model 2 added diabetes risk factors. The association between campaign exposure and DPP enrollment likelihood ratings decreased slightly but remained significant (β=.67, *P*<.001). Significant predictors included male gender (β=.28, *P=*.049), family history of diabetes (β=.33, *P=*.02), self-perceived overweight status (β=.47, *P=*.001), physical inactivity (β=.28, *P=*.047), and prediabetes diagnosis (β=.37, *P=*.02). Age and insurance type were no longer significant. History of gestational diabetes and hypertension were not significantly associated with DPP enrollment likelihood ratings.

Model 3 further adjusted for intrinsic factors. Campaign exposure remained significant, albeit slightly reduced (β=.52, *P*<.001). Significant predictors included male gender (β=.33, *P*=.01), residence outside Honolulu County (β=.31, *P*=.02), motivation index (β=.38, *P*<.001), inevitability belief (β=.20*, P*<.001), and perceived effectiveness of DPP (“improve a little,” β=.76; “improve a lot,” β=1.63, *P*<.001). Diabetes risk factors were no longer significant.

No multicollinearity was detected (variance inflation factor <2). The variance explained by the models increased across model 1 to model 3 but remained low (*R*^2^=0.08, 0.13, and 0.23, respectively). Graphical assessment of model assumptions indicated slight deviation from the normality assumption for model 1. Sensitivity analyses using ordered logistic regression showed similar results (not shown).

Table 3 presents the results of the subgroup analyses by race or ethnicity using model 3. Campaign exposure was positively associated with the outcome DPP enrollment likelihood ratings among NHOPI and other racial or ethnic groups, but the association did not reach statistical significance among Filipino adults. Notably, the estimated effect for the Filipino subgroup was larger in magnitude than those observed for NHOPI and other racial or ethnic groups and approached statistical significance. Across all subgroups, motivation index scores, inevitability belief, and perceived DPP effectiveness were consistently significant predictors.

**Table 3.** Subgroup analysis—linear regression models on National Diabetes Prevention Program enrollment likelihood ratings stratified by race or ethnicity.

Variable	Filipino				NHOPI^a^				All other races or ethnicities			
	β	SE	95% CI	*P* value	β	SE	95% CI	*P* value	β	SE	95% CI	*P* value
Intercept	−2.35	1.87	−6.02 to 1.32	.21	−.74	1.05	−2.80 to 1.31	.48	−1.00	0.99	−2.94 to 0.94	.31
Exposed (ref^b^: no)				.09				.04				.01
Yes	.65	0.38	−0.09 to 1.40		.45	0.21	0.03 to 0.87		.45	0.17	0.11 to 0.79	
Age (years)	.00	0.02	−0.04 to 0.04	.95	.00	0.01	−0.03 to 0.03	.94	−.01	0.01	−0.03 to 0.01	.27
Gender (ref: women)				.51				.99				.003
Man	−.26	0.39	−1.03 to 0.51		.00	0.26	−0.50 to 0.50		.51	0.17	0.17 to 0.85	
Education (ref: HS^c^ or lower)				.53				.15				.58
Some college	−.53	0.55	−1.60 to 0.55		.19	0.27	−0.34 to 0.72		.29	0.29	−0.28 to 0.86	
College graduate	−.43	0.60	−1.60 to 0.73		.44	0.32	−0.19 to 1.06		.15	0.30	−0.45 to 0.74	
Post college graduate	.10	0.66	−1.21 to 1.40		.88	0.40	0.10 to 1.66		.36	0.32	−0.27 to 0.99	
Household income, US dollars (ref: <$25,000)				.28				.77				.85
$25,000-$50,000	1.08	0.76	−0.41 to 2.57		−.19	0.37	−0.91 to 0.53		.18	0.34	−0.49 to 0.84	
$50,001-$100,000	.93	0.78	−0.61 to 2.46		−.29	0.38	−1.04 to 0.46		−.06	0.32	−0.68 to 0.56	
$100,001-$150,000	.11	0.82	−1.50 to 1.72		.11	0.46	−0.79 to 1.01		−.25	0.35	−0.92 to 0.43	
>$150,001	1.03	0.93	−0.79 to 2.86		−.44	0.49	−1.40 to 0.51		−.18	0.36	−0.89 to 0.53	
Don’t know/missing	1.15	0.94	−0.68 to 2.98		−.32	0.58	−1.46 to 0.82		−.08	0.50	−1.06 to 0.90	
Insurance type (ref: private)				.95				.001				.44
Medicare	−.52	1.68	−3.81 to 2.76		1.48	0.43	0.63 to 2.33		−.43	0.32	−1.07 to 0.20	
Medicaid	−.01	0.51	−1.01 to 0.98		.03	0.32	−0.59 to 0.65		−.50	0.30	−1.09 to 0.09	
TRICARE/Other	−.42	0.58	−1.56 to 0.73		.72	0.51	−0.28 to 1.71		−.05	0.25	−0.54 to 0.44	
None/missing	.06	0.94	−1.78 to 1.90		−.13	0.56	−1.23 to 0.96		−.33	0.39	−1.10 to 0.44	
County (ref: Honolulu County)				.08				.18				.21
All other counties	.80	0.45	−0.08 to 1.68		.32	0.23	−0.14 to 0.77		.24	0.19	−0.14 to 0.62	
Diabetes risk factors												
Family history	−.68	0.38	−1.42 to 0.06	.07	.17	0.26	−0.32 to 0.67	.49	.17	0.18	−0.17 to 0.52	.33
Overweight perception	−.03	0.37	−0.76 to 0.69	.93	.41	0.24	−0.05 to 0.87	.08	.17	0.19	−0.20 to 0.54	.37
Not physically active (<3x per week)	.65	0.40	−0.12 to 1.43	.10	.05	0.22	−0.38 to 0.49	.81	.25	0.19	−0.11 to 0.61	.18
Gestational diabetes	−.77	0.65	−2.04 to 0.49	.23	−.18	0.34	−0.84 to 0.48	.59	.29	0.33	−0.35 to 0.94	.37
Diagnosed with high blood pressure	.33	0.35	−0.36 to 1.02	.35	−.08	0.23	−0.54 to 0.38	.72	−.26	0.20	−0.65 to 0.13	.19
Diagnosed with prediabetes	.20	0.40	−0.58 to 0.99	.61	−.05	0.26	−0.55 to 0.45	.84	.36	0.22	−0.07 to 0.78	.10
Motivation index	.64	0.19	0.27 to 1.01	.001	.35	0.11	0.13 to 0.57	.002	.39	0.10	0.19 to 0.59	<.001
Inevitability belief	.15	0.09	−0.03 to 0.33	.11	.20	0.05	0.10 to 0.30	<.001	.20	0.05	0.11 to 0.29	<.001
Perceived DPP^d^ effectiveness (ref: decline/stay the same)				<.001				<.001				<.001
Don’t know	−.55	0.82	−2.15 to 1.04		.32	0.43	−0.52 to 1.15		.61	0.36	−0.10 to 1.32	
Improve a little	.14	0.64	−1.11 to 1.38		.50	0.37	−0.23 to 1.22		1.00	0.28	0.46 to 1.54	
Improve a lot	1.73	0.65	0.46 to 3.00		1.29	0.36	0.60 to 1.99		1.77	0.29	1.21 to 2.33	

a NHOPI: Native Hawaiian and Other Pacific Islander.

b Ref: reference category.

c HS: high school.

d DPP: National Diabetes Prevention Program.

Table 4 presents the results of the sensitivity analysis restricted to participants with prediabetes to better assess the robustness of the main findings. In this restricted sample, campaign exposure was not significantly associated with the outcome, and the magnitude of the estimated effect was attenuated (β=.30, *P*=.27). However, several key predictors remained statistically significant, including male gender (β=.72, *P*=.02), motivation index (β=.39, *P*=.02), inevitability belief (*β*=.16, *P*=.03), and perceived effectiveness of DPP (“improve a little,” β=1.19; “improve a lot,” β=2.28, *P*<.001).

**Table 4.** Sensitivity analysis—linear regression models on National Diabetes Prevention Program enrollment likelihood ratings among participants diagnosed with prediabetes.

Variable	β	SE	95% CI	*P* value
Intercept	−1.12	1.90	−4.84 to 2.60	.56
Exposed (ref^a^: no)				.27
Yes	.30	0.27	−0.23 to 0.84	
Age (years)	.00	0.02	−0.04 to 0.03	.84
Gender (ref: women)				.02
Man	.72	0.30	0.12 to 1.31	
Race or ethnicity (ref: Filipino)				.91
NHOPI^b^	.14	0.40	−0.65 to 0.93	
Other	.17	0.39	−0.60 to 0.95	
Education (ref: HS^c^ or lower)				.51
Some college	−.20	0.44	−1.07 to 0.66	
College graduate	−.17	0.48	−1.10 to 0.77	
Post college graduate	.36	0.55	−0.71 to 1.42	
Household income, US dollars (ref: <$25,000)				.95
$25,000-$50,000	.31	0.56	−0.79 to 1.41	
$50,001-$100,000	.15	0.52	−0.86 to 1.16	
$100,001-$150,000	−.06	0.57	−1.18 to 1.07	
>$150,001	.29	0.61	−0.90 to 1.48	
Don’t know/missing	.07	0.82	−1.53 to 1.68	
Insurance type (ref: private)				.97
Medicare	.26	0.47	−0.66 to 1.18	
Medicaid	.32	0.54	−0.73 to 1.38	
TRICARE/Other	−.04	0.59	−1.18 to 1.11	
None/missing	.23	1.11	−1.95 to 2.42	
County (ref: Honolulu County)				.22
All other counties	.40	0.32	−0.24 to 1.03	
Diabetes risk factors				
Family history	−.12	0.36	−0.83 to 0.58	.73
Overweight perception	.23	0.34	−0.43 to 0.90	.49
Not physically active (<3x per week)	−.06	0.27	−0.60 to 0.48	.82
Gestational diabetes	−.39	0.39	−1.16 to 0.38	.32
Diagnosed with high blood pressure	−.32	0.28	−0.87 to 0.24	.26
Motivation index	.39	0.17	0.05 to 0.73	.02
Inevitability belief	.16	0.07	0.01 to 0.30	.03
Perceived DPP^d^ effectiveness (ref: decline/stay the same)				<.001
Don’t know	1.23	0.63	0.00 to 2.45	
Improve a little	1.19	0.49	0.23 to 2.15	
Improve a lot	2.28	0.46	1.38 to 3.18	

a Ref: reference category.

b NHOPI: Native Hawaiian and Other Pacific Islander.

c HS: high school.

d DPP: National Diabetes Prevention Program.

## Discussion

### Principal Findings

This evaluation found that exposure to the Beat Diabetes campaign was associated with higher ratings of likelihood to enroll in a DPP among those at risk for prediabetes, after adjusting for demographic and other relevant factors. However, the campaign was not significantly associated with likelihood ratings among those medically diagnosed with prediabetes in the sensitivity analysis, which highlights the limits of including people with any risk of prediabetes in the cross-sectional study, not just those at highest risk. Despite this limitation, these findings show that a campaign with clear, action-oriented messaging may serve as a useful public health approach for targeting the approximately 80% of people at risk for prediabetes but unaware of their status [26].

The campaign builds on prior HDOH efforts, including the Prevent Diabetes*,* Hawai’i campaign, which focused on raising awareness of prediabetes among Native Hawaiian and Filipino adults [27]. Beat Diabetes extended this work by targeting a broader group disproportionately affected by diabetes and by emphasizing an immediate behavioral step—enrolling in a DPP. Although the campaign was designed to resonate among NHOPI or Filipino adults by featuring only NHOPI or Filipino individuals in all 7 advertisements, these groups were not more likely to report exposure, and their DPP enrollment likelihood ratings did not differ significantly from all other groups. In the subgroup analysis stratified by race or ethnicity, campaign exposure was statistically significant for NHOPI and all other races or ethnicities, while it was close to significant for Filipino adults. However, only 34 Filipino adults recalled the campaign, so it is possible that with a larger sample size, their exposure to the campaign could result with statistical significance. Additionally, grouping diverse races or ethnicities may have masked some campaign effects. Furthermore, the “other” group represents a combination of the remaining races or ethnicities outside of NHOPI and Filipino adults, including races or ethnicities with both high and low risk of prediabetes, such as African American and White adults, respectively. Aggregating them into a single group was important to explore the campaign exposure and likelihood ratings among the target populations of NHOPI and Filipino adults. Larger studies that enable disaggregation by race or ethnicity are needed to determine whether tailored messaging resonates more effectively within these target populations.

The regression models highlight the complex relationship between diabetes risk awareness and likelihood to enroll in a DPP. In model 2, several diabetes risk factors (family history, overweight perception, physical activity, and prediabetes diagnosis) were associated with higher likelihood ratings. However, in model 3, these were no longer significantly associated with likelihood to enroll in DPP. All 3 intrinsic factors—motivation, inevitability belief, and belief in DPP effectiveness—remained significantly associated with DPP enrollment likelihood ratings. This aligns with previous research that shows how additional factors, such as motivational or cognitive supports, may be necessary to translate contemplation to action [12,28]. Furthermore, belief in DPP effectiveness emerged as one of the strongest drivers, surpassing even the impact of campaign exposure. Participants who believed that DPP would improve their health “a little” had DPP enrollment likelihood ratings 0.75 points higher, and those who believed that it would improve their health “a lot” had ratings 1.63 points higher, compared with those who believed that DPP would have a neutral or negative effect. These differences were larger than the effect of campaign exposure (0.52-point increase), underscoring that confidence in DPP’s benefits strongly influences willingness to enroll. The sensitivity analysis of model 3 among those with a prediabetes diagnosis further supports this finding. It showed that the perception that DPP is effective was the most significant factor associated with likelihood to enroll in a DPP. In fact, among those with a prediabetes diagnosis who believed that DPP would improve their health “a lot” had a 2.28-point higher likelihood rating than those who thought that DPP would have no effect on their health and those who thought their health would decline. These findings are consistent with those of Joiner and colleagues [29], who reported that promoting the benefits of lifestyle interventions increased DPP enrollment among adults with prediabetes. Future campaigns may benefit from spotlighting participant success stories and emphasizing tangible health improvements associated with DPP participation.

Interestingly, in the main analysis and sensitivity analysis, inevitability belief scores were significantly associated with DPP enrollment likelihood ratings. Participants who believed that their diabetes progression was inevitable had higher DPP enrollment likelihood ratings. This finding was unexpected and counterintuitive based on findings by Ross et al [19], who interviewed a diverse set of British adults who were diagnosed with nondiabetic hyperglycemia and found that those who viewed diabetes progression as inevitable were less likely to pursue behavior change and questioned DPP’s value. It is unclear whether differences in study design or confounding are the source of the diverging findings. Furthermore, with a cross-sectional study design, it is unknown whether being aware of one’s diabetes risk influences feelings of inevitability of diabetes progression or whether feelings of inevitability influence one’s diabetes risk awareness. Both should be explored outside of this study design to fully understand whether a campaign can influence intention to enroll in DPP.

Individuals who identify as men reported higher DPP enrollment likelihood ratings, even in sensitivity analyses among those with diagnosed prediabetes. It is possible that men enrolled in the study just had higher baseline likelihood to enroll in DPP. It is also important to note that only 34.5% (293/860) of the sample identified as men, and of those men, only 42% (123/293) were exposed to the campaign. Given that men historically have had lower DPP participation rates [8], the association between gender and intention warrants future evaluation studies using more rigorous study designs and enrolling a higher portion of men, in particular, men of NHOPI or Filipino race or ethnicity, to explore whether media campaigns can be effective at changing intentions to enroll among men.

Despite these significant associations, the models’ fit statistics were relatively low, indicating that additional unmeasured factors likely play a major role in shaping enrollment intentions. Extrinsic structural and logistical barriers, such as the time commitment required for DPP or caregiving conflicts, have been well documented as barriers to DPP participation [12,17] but were not captured in this evaluation. This evaluation shows that for population-based efforts to reach all people with prediabetes risk, a campaign to encourage enrollment in DPP is only one small piece of the puzzle. A multifaceted strategy that pairs motivational messaging (eg, highlighting program effectiveness and success stories) with practical supports (eg, flexible schedules, virtual delivery, and culturally tailored programming) may be necessary to convert intention into enrollment for those at risk for prediabetes and those with a prediabetes diagnosis. Together, these findings help clarify which types of messaging may be most effective in increasing interest in DPP and point to the importance of pairing motivational communication with practical supports to translate intention into action.

### Limitations

This evaluation has several limitations. First, the cross-sectional study design can show only associations between DPP enrollment likelihood ratings and advertisement recall and not definitive causal impacts of the campaign. Also, without baseline data, it is impossible to know whether the associations observed between campaign recall and likelihood ratings are true or due to confounding. Individuals who saw the campaign may simply have higher baseline intentions to join a lifestyle change program or may have been more likely to recall the advertisement due to their higher perceived personal risk of diabetes, or for reasons unrelated to the campaign. Despite the inherent limitations of a cross-sectional study design, they are the most cost-effective and feasible method of evaluating the real-world implementation of a health department media campaign [24]. Second, the sample was drawn from market research panels, which comprised both consumer data and participants with interest and time to participate in market research. Regardless of efforts by the market research firm to ensure a diverse database representative of the state’s population and efforts to recruit participants from each county, the use of consumer panels limits the generalizability of the findings to the state’s population. Third, although the survey incentive was of a nominal amount appropriate for a 10‐ to 12-minute survey, incentives can bias participation, encouraging more participation by those of lower rather than higher socioeconomic status or enticing participants to repeatedly complete a survey to receive multiple incentives. Although our sample included participants from a range of household incomes, over half of the sample (513/860, 59.6%) had household incomes lower than Hawai’i’s median household income of US $100,745 [30], indicating the generalizability to the state’s population is limited. Also, to avoid repeated participation, the market research firm used various data integrity checks, such as removing responses from duplicate IP addresses and removing responses that were completed too quickly. Fourth, because the purpose of the study was to evaluate a statewide media campaign aimed at reaching as many people at risk for developing diabetes as possible, the inclusion criterion was any individual with at least 1 recognized prediabetes risk factor. This resulted in a sample that included individuals with very low absolute risk of developing diabetes (eg, only being aged 40 years or older), which limits the generalizability of the data to only population-based strategies aiming to reach large segments of the population, not studies aiming to reach only those at highest risk of diabetes. Finally, the evaluation relied on self-reported likelihood to join a DPP rather than actual enrollment behavior. It is unclear whether higher DPP enrollment likelihood ratings will translate into real-world participation. During survey development, HHET looked for a psychometrically validated survey measure of campaign impact on likelihood to join a DPP, but none existed. The closest validated measure was an 11-item survey scale measuring autonomous and controlled motivation [18], which did not fully align with the campaign’s goal of encouraging people to take urgent action. As a result, HHET developed a custom DPP enrollment likelihood question to measure intentions to change. However, because this measure is not psychometrically validated, its ability to accurately capture true likelihood to enroll is uncertain. Psychometric testing is warranted in the future to establish the validity of this item for measuring true likelihood to enroll in a DPP.

### Conclusions

The Beat Diabetes media campaign was designed to encourage people at risk for diabetes to take immediate action by joining a DPP. The campaign focused on NHOPI or Filipino adults who are disproportionately affected by prediabetes and diabetes in Hawai’i. However, the cross-sectional design of the study used a posttest survey that could observe associations only between campaign exposure and the likelihood or intent to join DPP. Results showed that NHOPI or Filipino adults did not report higher DPP enrollment likelihood ratings. Overall exposure was associated with higher likelihood ratings after controlling for multiple factors such as demographic characteristics, diabetes risk factors, and intrinsic motivations and beliefs. This association indicates that the campaign may be effective at encouraging people to consider joining a DPP. However, increasing one’s likelihood is the first step in the pathway to actual enrollment, and further evaluation is needed to determine whether media campaigns boost DPP enrollment and participation. Future campaigns aiming to boost DPP participation could be strengthened by promoting DPP effectiveness to increase DPP enrollment likelihood and emphasizing the urgency of taking preventive action at the prediabetes stage. More importantly, using motivational messaging alone is insufficient without addressing structural and logistical barriers to accessing DPPs.

## Supplementary material

10.2196/90880 Multimedia Appendix 1 A sample of still advertisements featured in the Beat Diabetes media campaign that ran from July to October 2024.

10.2196/90880 Multimedia Appendix 2 Postcampaign cross-sectional online survey conducted from October to December 2024.

10.2196/90880 Checklist 1 STROBE (Strengthening the Reporting of Observational Studies in Epidemiology) checklist.

10.2196/90880 Checklist 2 CHERRIES (Checklist for Reporting Results of Internet E-Surveys) checklist.

## Abbreviations

CHERRIES: Checklist for Reporting Results of Internet E-Surveys
DPP: National Diabetes Prevention Program
HDOH: Hawai'i State Department of Health
HHET: Healthy Hawai'i Evaluation Team
NHOPI: Native Hawaiian and Other Pacific Islander
STROBE: Strengthening the Reporting of Observational Studies in Epidemiology
