# Supplementary figures and images for: Encouraging Adults at Risk for Type 2 Diabetes to Enroll in Diabetes Prevention Programs Through a Media Campaign in Hawai’i: Cross-Sectional Study
Source: JMIR Public Health Surveill. 2026 Jun 18;12:e90880. doi: 10.2196/90880 (PMC13277823; doi:10.2196/90880)

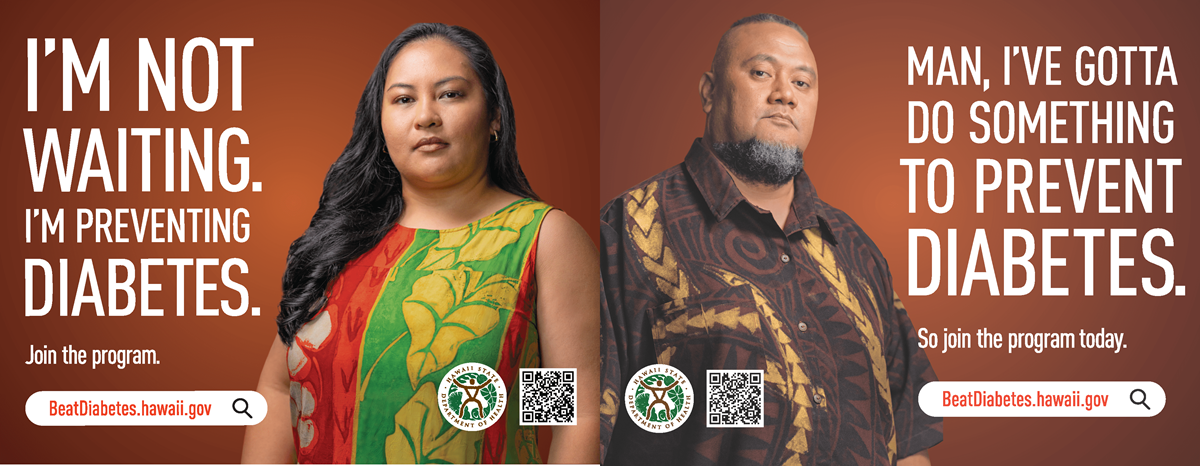

Supplement: Multimedia Appendix 1 [file publichealth-v12-e90880-s001.png]
