# Supplementary material for: Encouraging Adults at Risk for Type 2 Diabetes to Enroll in Diabetes Prevention Programs Through a Media Campaign in Hawai’i: Cross-Sectional Study
Source: JMIR Public Health Surveill. 2026 Jun 18;12:e90880. doi: 10.2196/90880 (PMC13277823; doi:10.2196/90880)
Supplement: Multimedia Appendix 2 [file publichealth-v12-e90880-s002.docx]

**Screening Questions:**

S1. Are you a full-time resident of Hawai‘i, living here at least six months per year?

- Yes
- No [TERMINATE]

S2. Have you ever been diagnosed with type 1 or type 2 diabetes?

- Yes [TERMINATE]
- No

S3. What was your age on your last birthday?

[fill in] years

- If younger than 35 [TERMINATE]
- If older than 64 [TERMINATE]

S4. Do any of the following apply to you? *(mark all that apply)*:

- My family members or relatives have diabetes
- I am physically active less than 3 times per week
- I have given birth to a baby that weighed more than 9 pounds
- I had gestational diabetes in a past pregnancy
- My doctor or other health professional told me that I have prediabetes or borderline diabetes
- My doctor or other health professional told me that I have high blood pressure
- None of these applies to me

S5. What would best describe your weight?

- Underweight [if under 40 yrs and S4= none, then TERMINATE]
- Normal weight [if under 40 yrs and S4= none, then TERMINATE]
- Overweight

S6. Do you identify as any of these ethnicities: Filipino, Native Hawaiian/Part Hawaiian, Other Pacific Islander (including Samoan, Tongan, Guamanian/Chamorro, Fijian and peoples of Micronesia and Melanesia)?

- - - - Yes
      - No

S7. What island do you live on?

- - - - Kauaʻi
      - Oʻahu
      - Maui
      - Molokaʻi
      - Lānaʻi
      - Hawaiʻi Island (Big Island)

[Introductory text for those who advance past the screening questions]

Aloha,

The Hawai‘i State Department of Health invites you to participate in an important online survey about diabetes prevention. Your responses will help us plan for program improvements that we hope will lead to better health for the people of Hawai‘i. To assist us with this project, we have partnered with Omnitrak Group, Inc, a Honolulu-based company.

The survey will consist of about 47 questions and will take approximately 10-12 minutes to complete. The survey is voluntary, and you can stop at any time. All survey responses will be confidential and will not include your personal information.

As a thank you for your participation, we would like to offer you a $10 Amazon e-gift card. You must qualify (some may not qualify) and complete the entire survey to be eligible for the e-gift card. 

If you have any questions about this project, please call or email Gail Ogawa at 808-586-4526 and [Gail.Ogawa@doh.hawaii.gov](http://doh.hawaii.gov/). Mahalo for your valuable feedback.

By clicking “Next” to start the survey, you are consenting to participate in the Diabetes Prevention Survey.

**Survey Questions:**

**SECTION 1: FEELINGS ABOUT YOUR HEALTH**

*Please answer the following questions on a scale of 1- Not at all true to 7- Very True, starting with:*

*The reason I would take steps to help prevent diabetes is that…*

Q1. It is very important for being as healthy as possible.

Not at all true (1) 🡪 Very true (7)

Q2. I personally believe it is the best thing for my health.

Not at all true (1) 🡪 Very true (7)

Q3. I feel that I want to take responsibility for my own health.

Not at all true (1) 🡪 Very true (7)

Q4. It is consistent with my life goals.

Not at all true (1) 🡪 Very true (7)

*The next three questions will ask about your thoughts about diabetes and your family history with the disease.*

Q5. It is only a matter of time before I get diabetes.

Not at all true (1) 🡪 Very true (7)

Q6. Does your family have a history of diabetes?

- Yes [go to Q7]
- No [SKIP LOGIC: go to Q8]

Q7. People in my family have diabetes so I will have it too.

Not at all true (1) 🡪 Very true (7)

Q8. Data integrity question: How many days per week are you physically active?

**SECTION 2: LIFESTYLE CHANGE PROGRAM**

*A lifestyle change program teaches healthy nutrition and exercise skills and has been shown by research to improve a person’s health. In the Beat Diabetes lifestyle change program, a lifestyle coach works with a group of people with prediabetes to develop the habits needed to lose weight and prevent diabetes.*

Q9. Based on this description, how likely are you to consider joining a lifestyle change program such as Beat Diabetes? Not at all likely (1) 🡪 Very likely (7)

Q10. If you joined a lifestyle program such as Beat Diabetes, do you think your health would:

- Decline a lot
- Decline a little
- Remain the same / Not change
- Improve a little
- Improve a lot
- Don’t know
- I prefer not to answer

**SECTION 3: DIABETES PREVENTION MEDIA CAMPAIGN ADS**

[UNAIDED Recall questions]

Q11. Between July 2024 and October 2024, did you see or hear any ads encouraging people to join a program to prevent diabetes in Hawaiʻi today and/or discouraging people to not put off joining a diabetes prevention program? These may have been on TV, radio, digital media, in newspapers, or at a shopping mall.

- Yes
- No [go to Q13]
- Don’t know/Not sure

Q12. What do you recall about the ad? [Open ended response]

[AIDED Recall questions]

*Now we’ll ask about some specific messages and advertisements encouraging people to join a diabetes prevention program right now/discouraging people to not put off joining a diabetes prevention program. You’ll see screen shots for each of the 4 print ads and one video ad. For each of the ads, please tell us if you remember seeing it or not. It’s okay if you haven’t seen all of these or even any of them.*

[randomize order of the recall questions for the different ads]


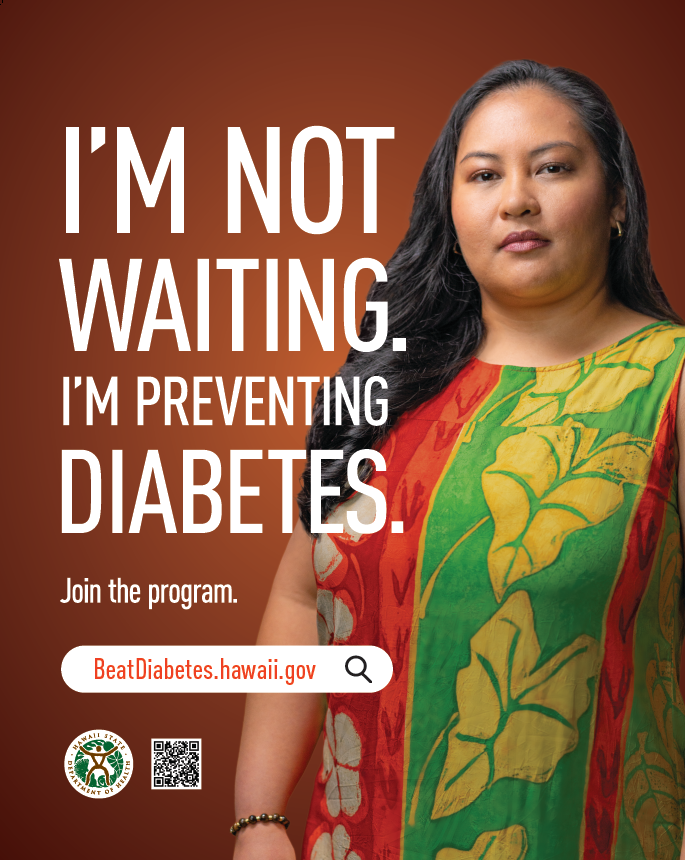


[Show Ad number 1 screenshot]

Q13. Have you seen or heard this ad with a woman saying that she is not waiting to prevent diabetes and will be joining a program?

- Yes
- No
- Don’t know/Not sure

Thinking about this ad with a woman saying that she is not waiting to prevent diabetes and will be joining a program, how would you rate the following:

Q14. The message tells me something important:

not at all true (1) 🡪 very true (7)

Q15. I feel the message was intended for people like me

not at all true (1) 🡪 very true (7)

Q16. This ad encourages me to do something to prevent diabetes now

not at all true (1) 🡪 very true (7)


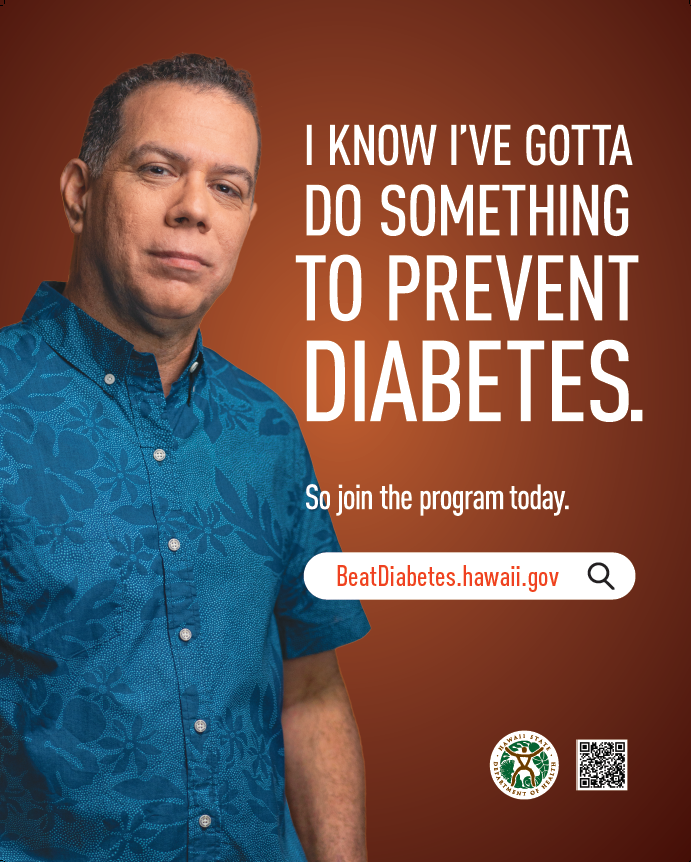


[Show Ad number 2 screenshot]

Q17. Have you seen or heard this ad of a man saying “he has gotta do something to prevent diabetes”?

- Yes
- No
- Don’t know/Not sure

Thinking about this ad with a man saying “he has gotta do something to prevent diabetes”, how would you rate the following:

Q18. The message tells me something important:

not at all true (1) 🡪 very true (7)

Q19. I feel the message was intended for people like me

not at all true (1) 🡪 very true (7)

Q20. This ad encourages me to do something to prevent diabetes now

not at all true (1) 🡪 very true (7)


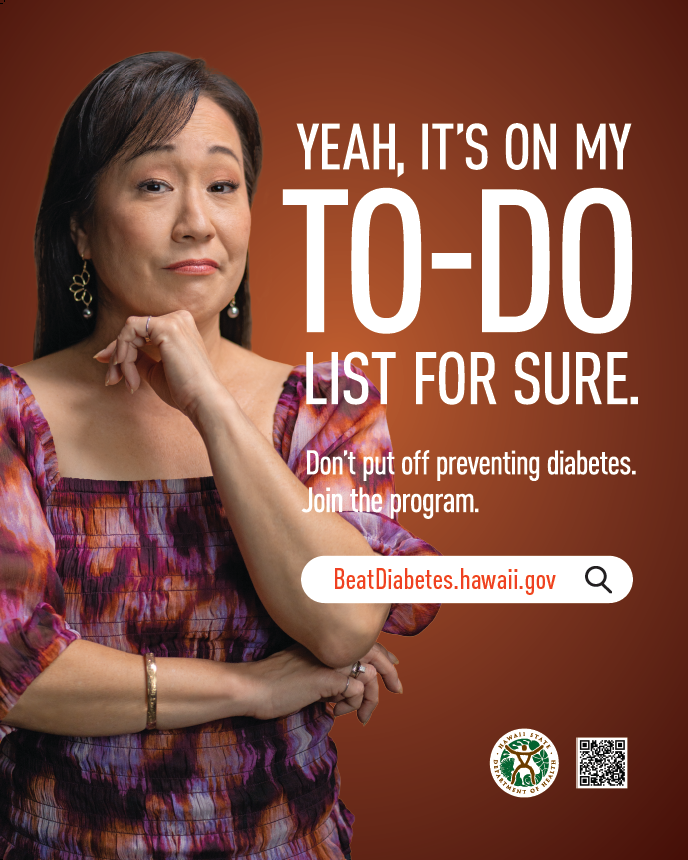


[Show Ad number 3 screenshot]

Q21. Have you seen this ad of a woman mentioning the diabetes prevention program being on “my TO-DO list”?

- Yes
- No
- Don’t know/Not sure

Thinking about this ad of a woman mentioning the diabetes prevention program being on “my TO-DO list”, how would you rate the following:

Q22. The message tells me something important:

not at all true (1) 🡪 very true (7)

Q23. I feel the message was intended for people like me

not at all true (1) 🡪 very true (7)

Q24. This ad encourages me to do something to prevent diabetes now

not at all true (1) 🡪 very true (7)

*
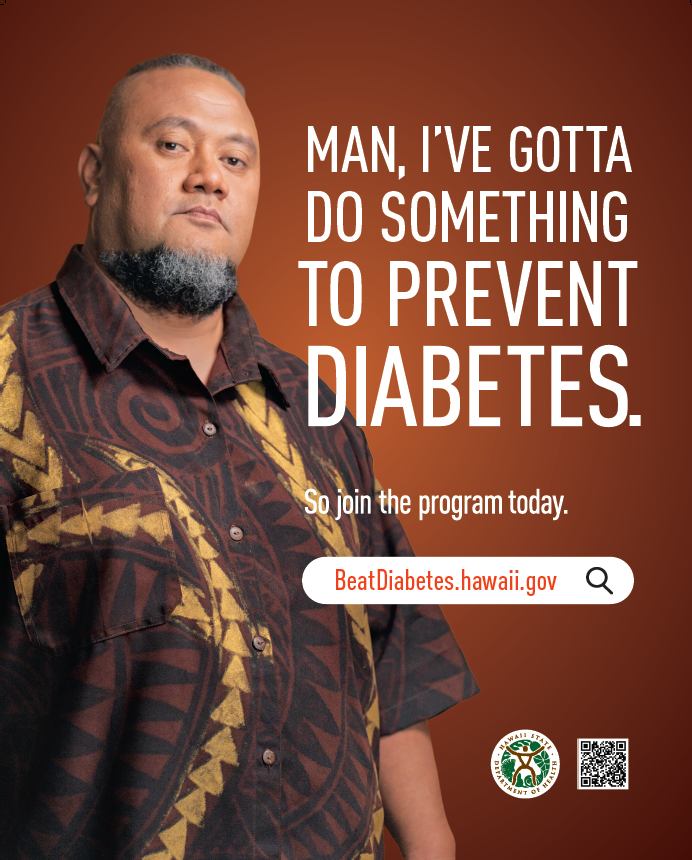
*

[Show Ad number 4 screenshot]

Q25. Have you seen this ad of a man saying he has gotta do something to prevent diabetes?

- Yes
- No
- Don’t know/Not sure

Thinking about this ad of a man saying he has gotta do something to prevent diabetes, how would you rate the following:

Q26. The message tells me something important:

not at all true (1) 🡪 very true (7)

Q27. I feel the message was intended for people like me

not at all true (1) 🡪 very true (7)

Q28. This ad encourages me to do something to prevent diabetes now

not at all true (1) 🡪 very true (7)


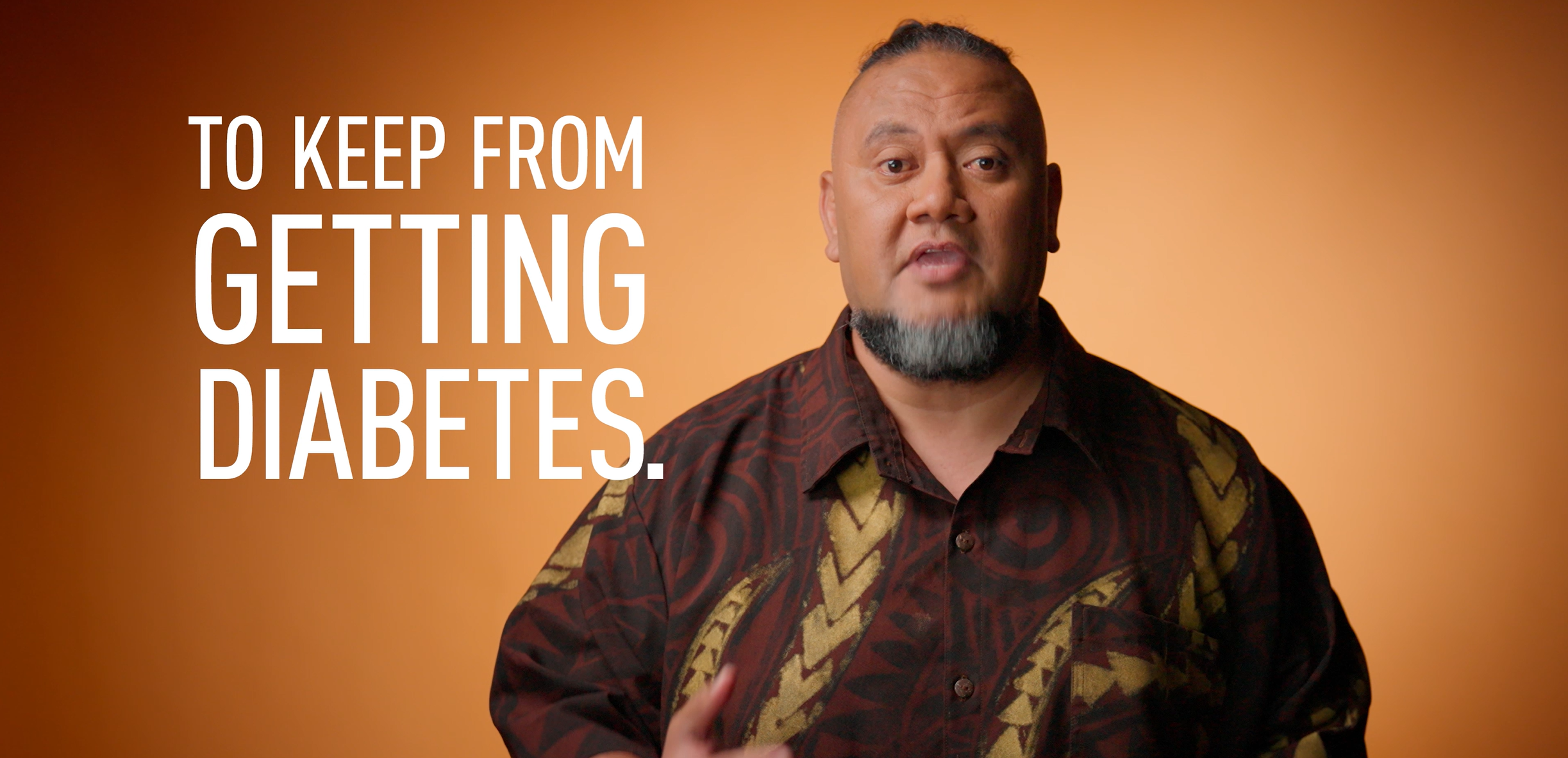


[Show Ad number 5 (video) - :30 pre-roll open caption. <https://youtu.be/KuV76upPHmM?si=OY4jEkZu4RlTtOYI>]

***Please click the play button to watch the following ad. Please watch the entire 30 second video before answering the question.***

Q29. Have you seen or heard this ad with multiple people saying they could not put off doing something to prevent getting diabetes, and then saying they joined the Beat Diabetes prevention program, and then the website is shown in the ad?

- Yes
- No
- Don’t know/Not sure

Thinking about the video ad you just watched, how would you rate the following:

Q30. The message tells me something important:

not at all true (1) 🡪 very true (7)

Q31. I feel the message was intended for people like me

not at all true (1) 🡪 very true (7)

Q32. This ad encourages me to do something to prevent diabetes now

not at all true (1) 🡪 very true (7)

[Ask Q33 only to those who said No to ALL aided recall questions: Q13, 17, 21, 25, and 29- **those who have NOT seen ANY of the specific ads]**

Q33. Did you see any of these other ads, a man saying Tomorrow’s a hard NOPE I’m preventing diabetes today and a woman saying Today I start preventing diabetes? [show screenshots of the remaining ads not shown in the random rotation in the previous questions]


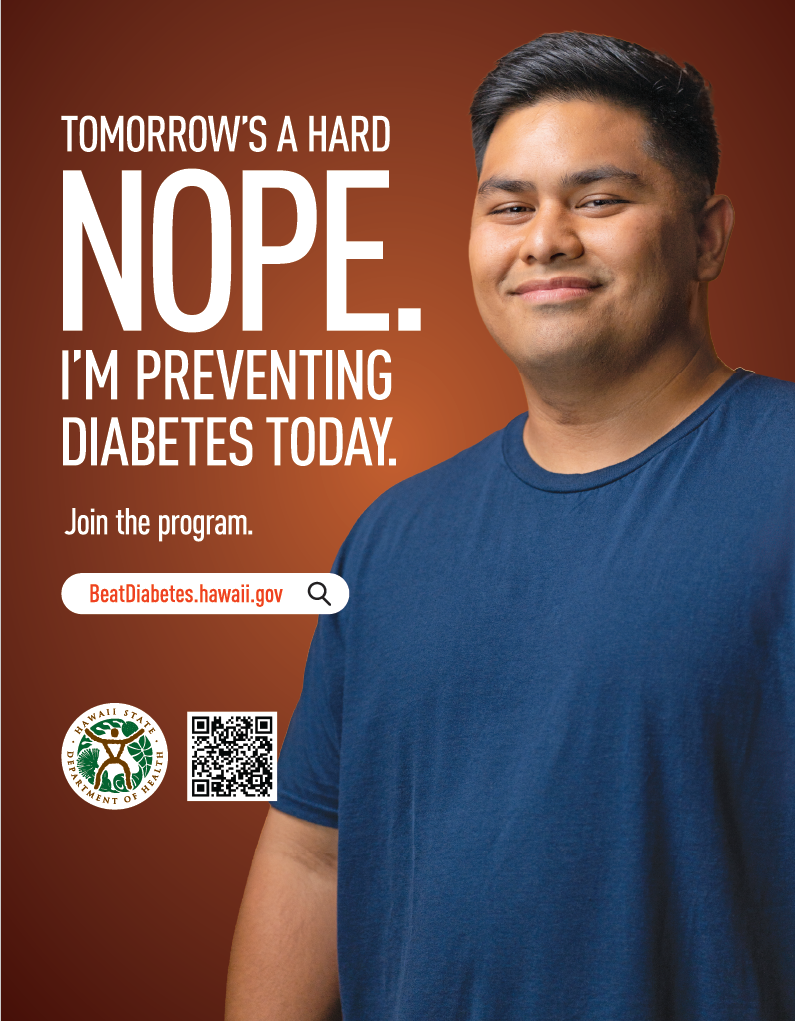

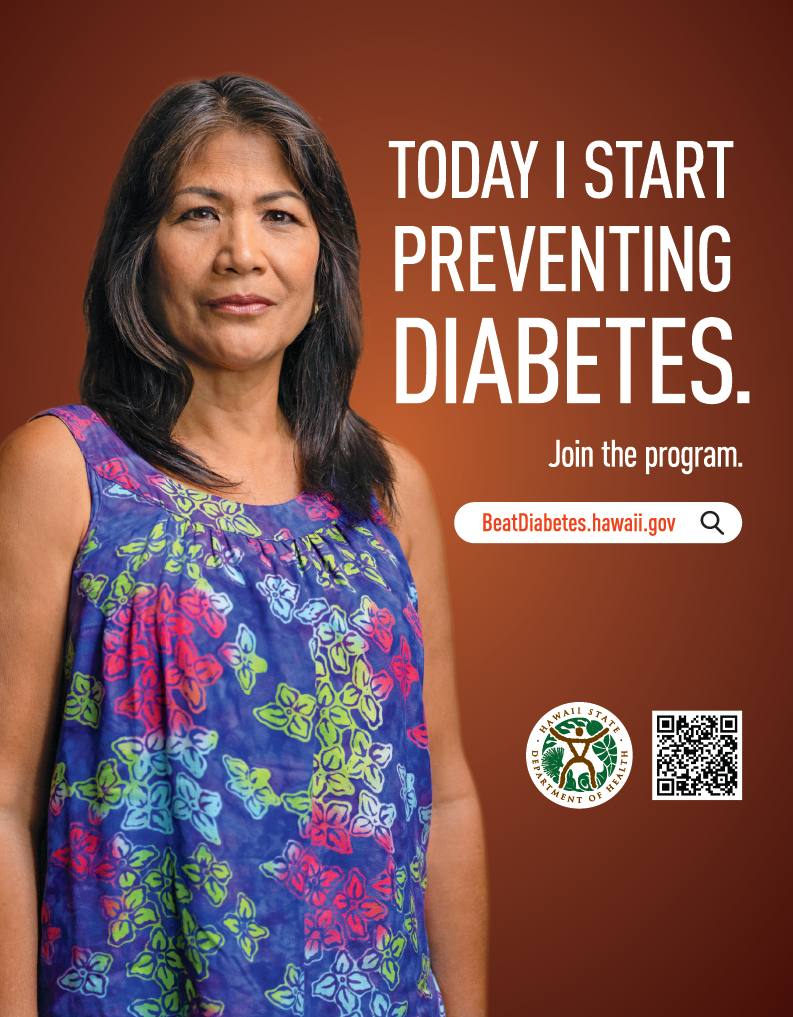


- Yes
- No [SKIP to Q37]

**SECTION 4: POST AD QUESTIONS [Q34-36 is for those who have seen the ads]**

*About all ads:* [If Yes to any of the ad recall questions above (Q13, 17, 21, 25, 29, or 33)]

Q34. How many times do you think you saw or heard these ads?

Once or twice

Every few weeks

Weekly

Multiple times per week

Don’t know/Not sure

Q35. Where do you recall seeing or hearing these ads? (*Select all that apply*)

- Facebook
- Instagram
- On a website
- Newspapers (e.g., Honolulu Star-Advertiser, MidWeek, The Garden Island, West Hawai‘i Today, Hawaiʻi Tribune-Herald)
- Television
- Radio
- Malls/Shopping Malls
- Other, please specify: [fill in]
- Don’t know/Not sure

Q36. What actions, if any, did you take after seeing these ads? *(mark all that apply)*

- I went to the Beat Diabetes website (BeatDiabetes.hawaii.gov)
- I filled out the intake form on the Beat Diabetes website (BeatDiabetes.hawaii.gov)
- I took a risk test to see if I have prediabetes
- I talked to family or friends about getting screened for prediabetes
- I discussed with a health provider about a lifestyle change program such as Beat Diabetes
- I asked a health provider for a referral to a lifestyle change program such as Beat Diabetes
- I enrolled in a lifestyle change program such as Beat Diabetes
- I thought about my risk for diabetes
- I learned about prediabetes on my own
- Other, please specify: [fill in]
- I didn’t take any action

[Q37 is only for those who did not see an ad until today- marked No to Q Q13, 17, 21, 25, 29, AND 33]

Q37. What actions, if any, do you think you are LIKELY to take after seeing these ads?

- Go to the website Beat Diabetes website (BeatDiabetes.hawaii.gov)
- Fill out the intake form on the Beat Diabetes website (BeatDiabetes.hawaii.gov)
- Take a risk test to see if I have prediabetes
- Talk to family or friends about getting screened for prediabetes
- Discuss with a health provider about a lifestyle change program such as Beat Diabetes
- Ask a health provider for a referral to a lifestyle change program such as Beat Diabetes
- Enroll in a lifestyle change program such as Beat Diabetes
- Think about my risk for diabetes
- Learn about prediabetes on my own
- Other, please specify: [fill in]
- Will not take action

**[The rest of the questions are for everyone]**

[Repeated MOTIVATION questions:]

Q38. Considering the messaging and advertisements you’ve seen regarding the Beat Diabetes prevention program, please answer the following questions asked earlier in this survey.

How likely are you to consider joining a lifestyle change program such as Beat Diabetes? 1=Not at all likely (1) 🡪 Very likely (7)

Q38a. Why did you choose a score of [value]?

*Please answer the following questions starting with “The reason I would take steps to help prevent diabetes is that:”*

Q39. Considering the messaging and advertisements you’ve seen regarding the Beat Diabetes prevention program, please answer the following questions asked earlier in this survey.

It is very important for being as healthy as possible.

Not at all true (1) 🡪 Very true (7)

Q40. I personally believe it is the best thing for my health.

Not at all true (1) 🡪 Very true (7)

Q41. I feel that I want to take responsibility for my own health.

Not at all true (1) 🡪 Very true (7)

Q42. It is consistent with my life goals.

Not at all true (1) 🡪 Very true (7)

**SECTION 5: DEMOGRAPHICS**

Q43. Which gender do you most identify with?

- Woman
- Man
- Transgender woman
- Transgender man
- Non-binary or Genderqueer
- Questioning
- Other, please specify: [fill in]
- I prefer not to answer

Q44. Which one or more of the following would you say is your race or ethnicity? *(mark all that apply)*

- American Indian or Alaska Native
- Black or African American
- Caucasian/White
- Chinese
- Filipino
- Guamanian or Chamorro
- Native Hawaiian/Part-Hawaiian
- Japanese
- Korean
- Other Asian
- Other Pacific Islander
- Hispanic/Latino/a/x
- Samoan
- Vietnamese
- Other, please specify: [fill in]
- I prefer not to answer

Q44a.Which one of these groups would you say best represents your race or ethnicity *(choose only one*)? [allow only one answer]

- American Indian or Alaska Native
- Black or African American
- Caucasian/White
- Chinese
- Filipino
- Guamanian or Chamorro
- Native Hawaiian/Part-Hawaiian
- Japanese
- Korean
- Other Asian
- Other Pacific Islander
- Hispanic/Latino/a/x
- Samoan
- Vietnamese
- Other: [fill in]
- I prefer not to answer

Q45. What is the highest level of education that you have completed?

- Less than high school degree
- High school degree or equivalent (e.g., GED)
- Some college or technical degree
- Associate’s degree
- Bachelor’s degree
- Graduate degree (e.g., Masters, PhD, MD)
- I prefer not to answer

Q46. What best describes your annual household income before taxes for 2023?

- Less than $25,000
- Over $25,000 to $35,000
- Over $35,000 to $50,000
- Over $50,000 to $75,000
- Over $75,000 to $100,000
- Over $100,000 to $150,000
- Over $150,000 to $200,000
- Over $200,000
- Don’t know
- I prefer not to answer

Q47. What is your primary source of health insurance?

- - Medicare
  - MedQuest
  - Tricare
  - Private insurance through an employer or union
  - Other, please specify: [fill in]
  - I don't have health insurance
  - I prefer not to answer

Thank you for your time. If you’re interested in learning more about preventing diabetes, visit BeatDiabetes.hawaii.gov. Please click the Submit button to submit your final responses and end the survey. (VENDOR TO INSERT NEXT STEPS REGARDING INCENTIVES)
