# Supplementary material for: Encouraging Adults at Risk for Type 2 Diabetes to Enroll in Diabetes Prevention Programs Through a Media Campaign in Hawai’i: Cross-Sectional Study
Source: JMIR Public Health Surveill. 2026 Jun 18;12:e90880. doi: 10.2196/90880 (PMC13277823; doi:10.2196/90880)
Supplement: Checklist 1 [file publichealth-v12-e90880-s003.docx]

STROBE Statement—Checklist of items that should be included in reports of ***cross-sectional studies***

|  | Item No | Recommendation | Location (or reason for not reporting) |
| --- | --- | --- | --- |
| **Title and abstract** | 1 | (*a*) Indicate the study’s design with a commonly used term in the title or the abstract | Title and first sentence of Methods section of Abstract |
|  |  | (*b*) Provide in the abstract an informative and balanced summary of what was done and what was found | Abstract: Methods, Results |
| Introduction | | | |
| Background/rationale | 2 | Explain the scientific background and rationale for the investigation being reported | First two sentences in Abstract: Background |
| Objectives | 3 | State specific objectives, including any prespecified hypotheses | Abstract: Objective and last paragraph of the Introduction section |
| Methods | | | |
| Study design | 4 | Present key elements of study design early in the paper | First paragraph of the Methods section: Participant Recruitment |
| Setting | 5 | Describe the setting, locations, and relevant dates, including periods of recruitment, exposure, follow-up, and data collection | First paragraph of the Methods section: Participant Recruitment |
| Participants | 6 | (*a*) Give the eligibility criteria, and the sources and methods of selection of participants | Sentence 6 & 7 in Methods: Participant Recruitment  Figure 1 |
| Variables | 7 | Clearly define all outcomes, exposures, predictors, potential confounders, and effect modifiers. Give diagnostic criteria, if applicable | Methods: Survey Measures and in Limitations and Future Directions |
| Data sources/ measurement | 8* | For each variable of interest, give sources of data and details of methods of assessment (measurement). Describe comparability of assessment methods if there is more than one group | Methods: Survey Measures |
| Bias | 9 | Describe any efforts to address potential sources of bias | Limitations and Future Directions |
| Study size | 10 | Explain how the study size was arrived at | Methods: Participant Recruitment |
| Quantitative variables | 11 | Explain how quantitative variables were handled in the analyses. If applicable, describe which groupings were chosen and why | Methods: Data Analysis |
| Statistical methods | 12 | (*a*) Describe all statistical methods, including those used to control for confounding | Methods: Data Analysis |
|  |  | (*b*) Describe any methods used to examine subgroups and interactions | Methods: Data Analysis |
|  |  | (*c*) Explain how missing data were addressed | Methods: Data Analysis |
|  |  | (*d*) If applicable, describe analytical methods taking account of sampling strategy | Methods: Participant Recruitment |
|  |  | (*e*) Describe any sensitivity analyses | Methods: Data Analysis, 2nd paragraph |
| Results | | | |
| Participants | 13* | (a) Report numbers of individuals at each stage of study—eg numbers potentially eligible, examined for eligibility, confirmed eligible, included in the study, completing follow-up, and analysed | Results: Final Sample; Figure 2 |
|  |  | (b) Give reasons for non-participation at each stage | Results: Final Sample; Figure 2 |
|  |  | (c) Consider use of a flow diagram | Figure 2 |
| Descriptive data | 14* | (a) Give characteristics of study participants (eg demographic, clinical, social) and information on exposures and potential confounders | Results: Sample Characteristics; Table 1 |
|  |  | (b) Indicate number of participants with missing data for each variable of interest | Footnote “b” in Table 1 |
| Outcome data | 15* | Report numbers of outcome events or summary measures | Results: Sample Characteristics by Campaign Exposure; Linear Regression Models on DPP Enrollment Likelihood Rating |
| Main results | 16 | (*a*) Give unadjusted estimates and, if applicable, confounder-adjusted estimates and their precision (eg, 95% confidence interval). Make clear which confounders were adjusted for and why they were included | Results: Linear Regression Models on DPP Enrollment Likelihood Rating; Tables 2-4 |
|  |  | (*b*) Report category boundaries when continuous variables were categorized | Not applicable |
|  |  | (*c*) If relevant, consider translating estimates of relative risk into absolute risk for a meaningful time period | Not relevant |
| Other analyses | 17 | Report other analyses done—eg analyses of subgroups and interactions, and sensitivity analyses | Results: Linear Regression Models on DPP Enrollment Likelihood Rating  Table 3 and 4 |
| Discussion | | | |
| Key results | 18 | Summarise key results with reference to study objectives | Discussion: Principal Findings, Paragraphs 1-8 |
| Limitations | 19 | Discuss limitations of the study, taking into account sources of potential bias or imprecision. Discuss both direction and magnitude of any potential bias | Discussion: Limitations and Future Directions |
| Interpretation | 20 | Give a cautious overall interpretation of results considering objectives, limitations, multiplicity of analyses, results from similar studies, and other relevant evidence | Discussion: Conclusions |
| Generalisability | 21 | Discuss the generalisability (external validity) of the study results | Discussion: Limitations and Future Directions, 2nd, 3rd , and 4th limitations |
| Other information | | | |
| Funding | 22 | Give the source of funding and the role of the funders for the present study and, if applicable, for the original study on which the present article is based | Discussion: Funding, paragraph 1 |

*Give information separately for exposed and unexposed groups.

**Note:** An Explanation and Elaboration article discusses each checklist item and gives methodological background and published examples of transparent reporting. The STROBE checklist is best used in conjunction with this article (freely available on the Web sites of PLoS Medicine at http://www.plosmedicine.org/, Annals of Internal Medicine at http://www.annals.org/, and Epidemiology at http://www.epidem.com/). Information on the STROBE Initiative is available at www.strobe-statement.org.
